# Astrocytes, neurons, synapses: a tripartite view on cortical circuit development

**DOI:** 10.1186/s13064-018-0104-y

**Published:** 2018-05-01

**Authors:** Isabella Farhy-Tselnicker, Nicola J. Allen

**Affiliations:** Molecular Neurobiology Laboratory, Salk Institute for Biological Studies, 10010 North Torrey Pines Rd, La Jolla, CA 92037 USA

**Keywords:** Astrocyte, Neuron, Synapse, Development, Cortex

## Abstract

In the mammalian cerebral cortex neurons are arranged in specific layers and form connections both within the cortex and with other brain regions, thus forming a complex mesh of specialized synaptic connections comprising distinct circuits. The correct establishment of these connections during development is crucial for the proper function of the brain. Astrocytes, a major type of glial cell, are important regulators of synapse formation and function during development. While neurogenesis precedes astrogenesis in the cortex, neuronal synapses only begin to form after astrocytes have been generated, concurrent with neuronal branching and process elaboration. Here we provide a combined overview of the developmental processes of synapse and circuit formation in the rodent cortex, emphasizing the timeline of both neuronal and astrocytic development and maturation. We further discuss the role of astrocytes at the synapse, focusing on astrocyte-synapse contact and the role of synapse-related proteins in promoting formation of distinct cortical circuits.

## Background

The mammalian cerebral cortex is a complex brain structure, which coordinates sensory and motor information and enables the animal to perform complex tasks. Neurons in the cortex are arranged in defined layers, and communicate across these layers as well as with other cortical and subcortical areas [1–3]. This creates a highly complex network of neuronal connections comprising the different cortical circuits. To ensure proper brain function developing cortical neurons must find the right partner and form the right connections: the synapses, a crucial step in correct circuit formation.

Neuronal synapses are contact sites where signals between two neurons are transferred [4]. In a chemical synapse, information from the presynaptic terminal of one neuron is processed via release of neurotransmitters, which bind their respective receptors on the postsynaptic side of the second neuron, activating downstream signaling pathways [4]. While synaptic activity was recorded over a century ago using electrophysiology, it wasn’t until the 1950s, with development of electron microscopy, that synapse structures were visualized. It was then discovered that neuronal synapses are not just composed of pre and postsynaptic neurons, but in many cases are also contacted by an astrocyte process [5–7].

Astrocytes are a major type of glia, a class of non-neuronal brain cells which also include oligodendrocytes, oligodendrocyte precursor cells (NG2 cells) and microglia [8]. For many years astrocytes were considered important, yet passive supporters of neurons, providing metabolic support, neurotransmitter precursors and ion buffering. Research demonstrated that astrocyte ablation in vivo or culturing neurons without astrocytes resulted in neuronal degeneration and ultimately death (reviewed in [9, 10]). Due to this inability of neurons to survive without astrocytes, the role of astrocytes in several aspects of neuronal function, such as synapse formation and activity was not assessed until more recently. Experiments using pure neuronal cultures, which were grown in conditions enabling them to survive in the absence of astrocytes [11, 12], demonstrated that astrocytes can actively promote formation of nascent neuronal synapses. Subsequent studies using in vitro and in vivo approaches discovered that astrocytes also regulate synapse maintenance and promote synapse elimination, thus regulating the overall architecture and activity of neuronal circuits and ultimately animal behavior. Astrocytes regulate synapses by direct contact [13–16], and by secreting soluble factors that target pre and postsynaptic sites, thereby modulating the structure and function of both excitatory and inhibitory synapses [12, 17–29]. This led to the concept of the “tripartite synapse”, a synapse composed of two neurons and an astrocyte as a functional unit [7]. In a tripartite synapse, the neurotransmitters released from neurons also bind receptors on the adjacent astrocyte process, activating signaling pathways in the astrocytes which modulate synaptic behavior [7, 30]. In addition to contacting neurons, astrocytes are interconnected with each other by gap junctions, specialized channels which allow nutrients and ions to diffuse between networks of astrocytes, expanding further the range and magnitude of synaptic regulation of neurons by astrocytes [31].

Much of the work on neuronal development, astrocyte development, synapse development and astrocyte regulation of synapse formation has been conducted by different groups, studying different model systems, brain areas and stages of development. This great body of work has led to many discoveries that have advanced our understanding of these processes. However, the diversity of model systems, brain regions and developmental stages studied can make it challenging to evaluate the in vivo contribution of astrocytes to synaptic development and maturation, in the context of ongoing neuronal and astrocyte development. In this review we synthesize this information in one place, and ask when do each of these developmental processes occur in the rodent cortex? We first summarize the stages of tripartite synapse development and circuit formation, starting from the generation of neurons and astrocytes, followed by the maturation of neuronal and astrocyte processes, and the developmental expression of key synaptic proteins in neurons and synaptogenic proteins in astrocytes. We use this foundation to ask questions about how astrocytes regulate synaptic development, including their role in promoting synaptic diversity and the formation of distinct cortical connectivity patterns. To read about the roles of astrocytes in other aspects of neuronal synapse function (i.e. maturation, elimination and plasticity) see the following reviews [9, 30, 32–34].

## The path from neurogenesis to synaptogenesis runs through astrogenesis

**Fig. 1:**
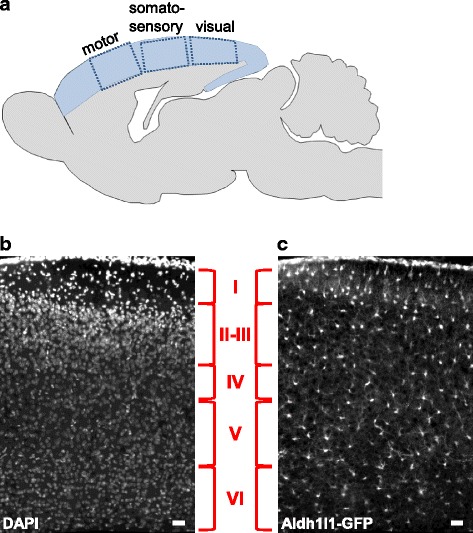
Overview of the cortex. **a** Schematic of the rodent brain section in sagittal orientation. Cerebral cortex is shaded in blue. Dashed boxes represent functional cortical areas as labeled. **b** P7 mouse visual cortex labeled with DAPI (white) to mark cell nuclei. **c** Same image as **b**, showing astrocyte marker Aldh1l1 (white), obtained from mice expressing GFP under the Aldh1l1 promoter. Cortical neurons are arranged in 6 layers, marked in red. Astrocytes are present in all cortical layers in the visual cortex. Scale bar = 50 μm

**Fig. 2:**
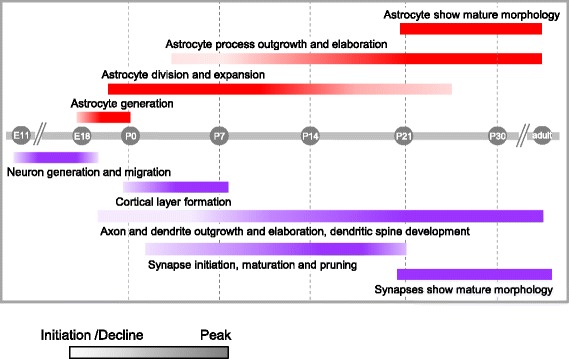
A combined overview of astrocyte, neuron and synapse generation and development. Timeline (grey) of key developmental processes in the rodent cortex from embryonic stages to the end of the first month of life, from neurogenesis, to astrogenesis to synapse formation, maturation and stabilization. Developmental processes as occur in astrocytes (red, above), and neurons (purple, below) are shown. Each process is represented as a colored bar, with the gradient of color intensity marking the beginning, peak, and end of the process

In this section we summarize key processes in the development of the rodent cortex (Fig. 1). We begin with the generation of neurons and their population of the cortex, which occurs before birth in the mouse (the average gestation period in mice is 18 days [35]). We will then overview the process of astrocyte generation, which begins at birth and continues through the first two postnatal weeks (Fig. 2). Finally we discuss the importance of astrocytes in synapse formation, which occurs in the first two postnatal weeks concurrent with the generation and maturation of astrocytes. For in depth reviews of neurogenesis and astrogenesis see [36–47].

### Step 1 - neurogenesis and establishment of neuronal connectivity

Neurons in the adult cortex are arranged into 6 horizontal layers and vertically into functional columns, receiving input from specific sensory areas, and interconnected in a highly specialized way to construct the mature cortical circuit [3, 38, 39] (Fig. 1). The generation of cortical neurons in mice begins at embryonic day (E) 10–11 following neural tube closure (E8-E9.5) [48]. Progenitor cells (also termed radial glia, RG) which are derived from neuroepithelial stem cells located in the ventricular zone (VZ) in the dorsal telencephalon undergo asymmetric division to give rise to post mitotic neurons which migrate outward to form the cortical plate (Fig. 1). RG also generate intermediate progenitors by symmetric division, which locate to the subventricular zone (SVZ) [49] and further differentiate to neurons (and later astrocytes and oligodendrocytes) [38, 47, 50, 51]. In addition, RG cells extend long processes which span the cortex and provide a scaffold for migrating neurons [47, 52]. The term radial glia was given to these progenitors due to their morphological similarity to immature glial fibers [53] and expression of several glial specific genes, such as glial fibrillary acidic protein (GFAP) and the glutamate transporter GLAST [47], but they are distinguished here from the mature class of glial cells. Neurons populate the cortex in an “inside out” pattern, where deep layer neurons are first to form, and superficial layer neurons are last to form [36, 54]. About 80% of neurons in the adult mouse cortex are excitatory pyramidal neurons, and the rest are a diverse population of inhibitory GABAergic interneurons [55, 56]. Inhibitory interneurons are generated from progenitors located in the medial and caudal ganglionic eminences (MGE and CGE) that migrate to populate the cortex at the time of neurogenesis (E11-P0) [57].

**Fig. 3:**
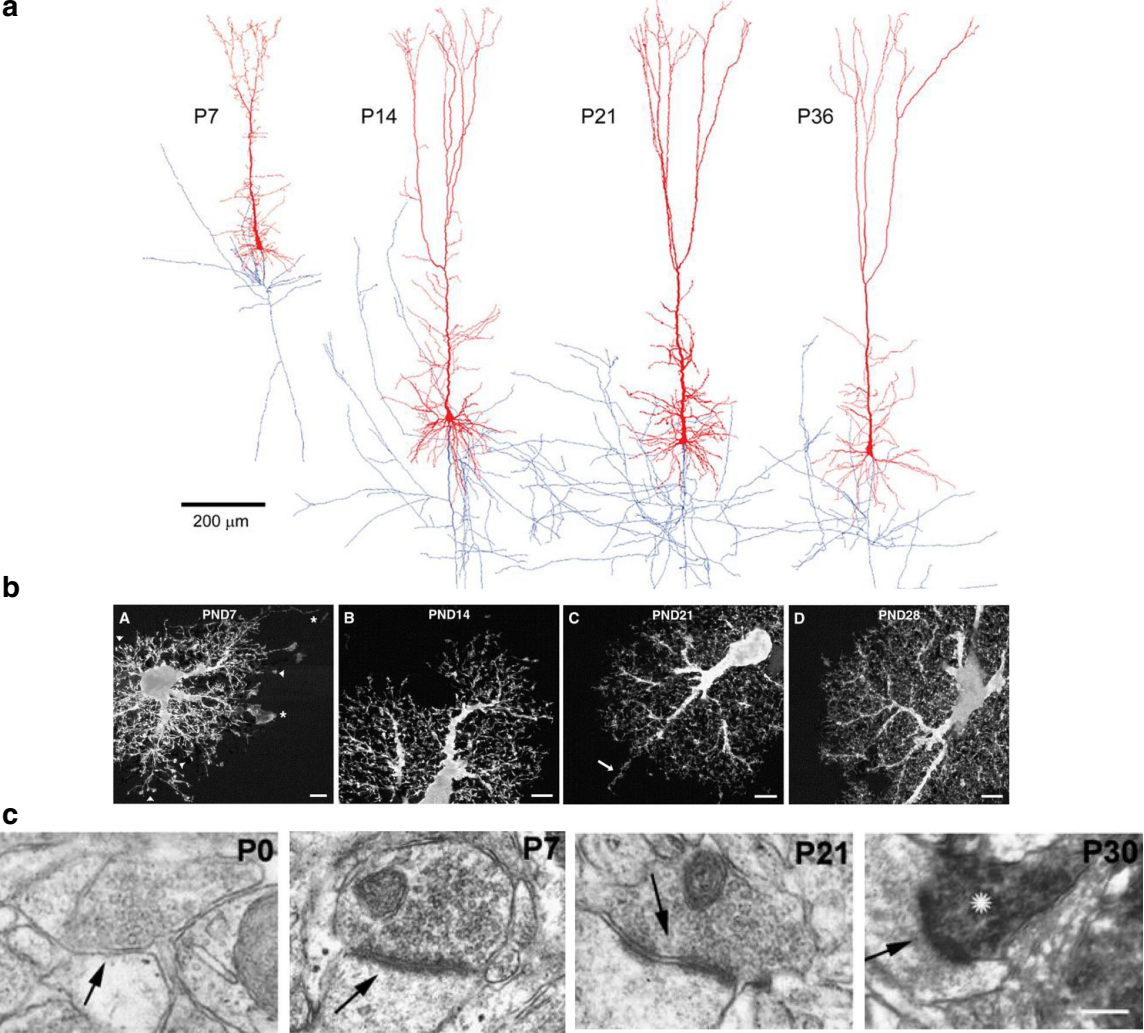
Neuronal and astrocytic process elaboration follows a similar timeline as synapse development. **a** Representative 3-D rendering of layer V rat SSC neurons reconstructed from biocytin-labeled neurons at different ages as labeled (dendrites in red, axons in blue). At P14, 21 and 36 the axons are shown cropped due to space limitations. Scale bar = 200 μm. Adapted with permission from [131]. **b** Representative images of Lucifer yellow filled rat hippocampal astrocytes at different developmental stages as indicated in each panel. Astrocyte process ramification increases with age. Scale bars = 5 μm. Adapted with permission from [67]. **c** Development of synapses in the mouse visual cortex visualized by electron microscopy at the different developmental ages as labeled. At P0 neurotransmitter vesicles can be visualized, but the postsynaptic density is not present. At P7, P21 and P30 presynaptic terminals with neurotransmitter vesicles apposed to postsynaptic density structures, marking synaptic contacts, are shown. Asterisk at P30 indicates immunoreactivity for the presynaptic marker synaptophysin. Scale bar = 130 nm. Adapted with permission from [84]

At birth (P0) cortical neurogenesis has finished, however late born neurons are still migrating to the upper layers and it isn’t until postnatal day (P)7 that the arrangement of neurons into defined cortical layers is completed and resembles the adult structure [54, 58]. Concurrent with neurogenesis and migration, neurons begin to establish interactions with each other, which will later evolve into synapses. The axons of newly generated neurons extend to find their future postsynaptic partners, and dendrites begin to form the protrusions that mark potential postsynaptic sites. For example, in the visual cortex, axons from thalamic neurons that originate in the dorsal lateral geniculate nucleus (dLGN) reach their post synaptic partners in layer IV between E15 and E18. At this time, cortical neurons from layers V and VI extend axons out towards their post synaptic targets in the dLGN [54]. Axons and dendrites continue to grow and mature throughout the first 2–3 postnatal weeks, reaching a mature morphology at the end of the first month (Figs. 2 and 3).

### Step 2 - Astrogenesis and astrocyte development

Following cortical neurogenesis (E18/P0 in rodents i.e. birth), astrocytes are generated from the same progenitor cells which gave rise to neurons [42, 47]. These progenitors undergo a potency switch from a neurogenic to a gliogenic differentiation program and differentiate into astrocytes. The mechanism for this switch involves activation of Notch1 [59] and Jak/STAT signaling pathways [60, 61] as well as the transcription factors sox9 and Nuclear factor 1A (NF1A) [62]. Activation of these pathways leads to de-methylation and promotion of expression of astrocyte specific genes, such as glial fibrillary acidic protein (GFAP) (reviewed in [43, 46]). Importantly, unlike the post mitotic neurons which populate the cortex after undergoing a terminal division, newborn astrocytes continue to divide locally after migration, and in this way generate half of the upper layer astrocytes [63]. In addition, upon completion of neuronal migration, the cortex spanning radial glia differentiate into astrocytes [49]. Astrocytes continue to expand in number through the end of the first month of life, and during this time take on a mature morphology [63, 64]. Similar to the growth and elaboration of neuronal processes (i.e. axons and dendritic arbors), during the first postnatal weeks there is extensive astrocyte process outgrowth (Figs. 2 and 3), and astrocytes develop their elaborate fine processes which come in contact with neuronal synapses. Towards the end of the third postnatal week excess astrocyte filopodia are pruned and astrocytes establish a tiled pattern, where each astrocyte occupies its own non-overlapping domain [65–68].

In the adult animal, astrocytes are present throughout all cortical areas and in all neuronal layers (Fig. 1). Interestingly, recent findings suggest that cortical astrocytes display a functional diversity as demonstrated by layer and region specific expression of synaptogenic factors [18, 69–72]. It is unclear if these diverse properties of astrocytes develop over time, or are intrinsic features of regionally developed astrocytes. Moreover, gap junction interconnected astrocytes are segregated between different functional cortical areas, such as neighboring columns in the barrel cortex [73, 74]. This suggests that within each functional cortical area, astrocytes are present as interconnected units, and can selectively respond to specific subsets of excitatory neurons [75]. It is therefore possible that, similar to neurons, cortical astrocytes are also arranged in functionally defined layers and/or columns. This is particularly interesting in the context of astrocyte modulation of specific synaptic connections (as discussed below). Since neurons in each layer of the cortex have distinct properties and connections, it will be interesting to test if astrocytes have layer-specific properties as well; for example, whether they specifically drive formation of either translaminar or columnar connections (or both) during development.

### Step 3 - Synaptogenesis – It takes both cell types to build a synapse

Although neurons send out projections before birth, synapses only begin to form during the first week of postnatal development, concurrent with the appearance of astrocytes [12, 41]. Multiple findings over the past years using in vitro neuronal cultures from retina and cortex have shown that neurons cultured in isolation make few synapses, and synapse formation is markedly increased upon addition of astrocytes or astrocyte-secreted factors. Using these cultures several astrocyte secreted proteins which promote formation of different types of excitatory glutamatergic synapses have been identified (for review see [9, 33]). Overall, these findings provide strong evidence for an active role of astrocytes in promoting synaptogenesis in vitro. In the following section we will describe the stages of synapse formation in the cortex in vivo, and how astrocytes may be regulating each stage. We examine the developmental timeline of neuronal and astrocyte development and maturation, concurrent with synapse development, as well as overview the developmental expression of synaptic proteins in both cell types. We will focus mainly on excitatory synapse formation as the majority of studies on astrocyte modulation of synapse formation were tested on these synapses. We will also briefly discuss inhibitory synapse formation and speculate on the roles of astrocytes in this process.

## Development of the cortical tripartite synapse

**Fig. 4:**
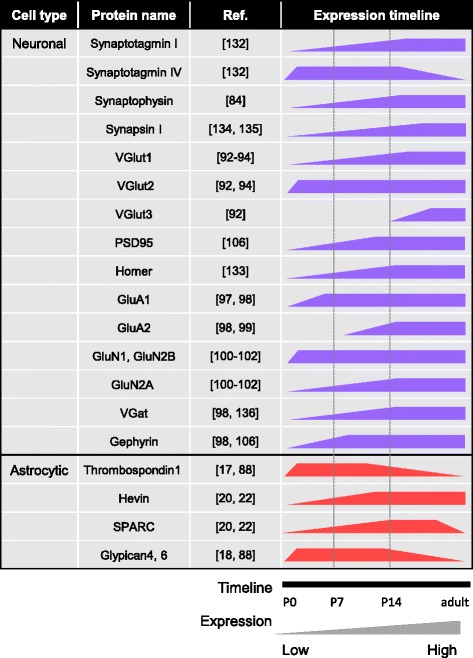
Timeline of expression of neuronal and astrocytic synapse-related proteins in the cortex [17, 88, 18, 20, 22, 84, 92–94, 97–99, 100–102, 106, 132–136]

Before we describe the different developmental stages of synaptogenesis, it is important to first determine what makes up a synapse at both structural and functional levels, and techniques used to study them. Synapses share common structural features which can be observed using imaging techniques such as electron microscopy (EM) (reviewed in [76, 77]). These include presynaptic terminals containing neurotransmitter vesicles, a post synaptic density where receptors are located, and in many cases, an astrocyte process, which can be identified using EM by its clear cytoplasm and the presence of glycogen granules and intermediate filament bundles [5, 6]. In addition, expression of synapse related proteins in both cell types can be measured using genomic and proteomic approaches. For example, immunofluorescence with specific antibodies against proteins expressed in either side of the neuronal synapse can be used to visualize the tripartite synapse [78, 79]. To visualize astrocyte processes tools to flourescently label astrocytes have been the most widely used method [68, 80, 81]. Additionally, several astrocyte specific proteins that are expressed in astrocyte processes and near synapses have been identified, such as glutamate transporters (GLAST and GLT1 [82]) or ion channels (such as Kir4.1 [83]), making it possible to use these as markers for the astrocyte part of the tripartite synapse. Neuronal synaptic activity can be measured using electrophysiology, while astrocyte responses can be visualized by imaging changes in astrocyte intracellular Ca^2+^ levels. By combining findings from experiments using these different approaches we can construct a time line of how synapses develop. By correlating this with astrocyte development and astrocyte expression of synapse promoting proteins at the same developmental stages, we can begin to extrapolate the specific roles of astrocytes in the different stages of synapse development (Figs. 2, 3 and 4).

### Timeline of synaptogenesis and expression of synaptic proteins by neurons and astrocytes

Formation of chemical synapses begins during the first postnatal week, peaks at P14, and stabilizes at P21 to P28, concurrent with synapse elimination and the refinement of circuits (Fig. 2). The earliest synaptic structures in the cortex, namely axonal terminals with presynaptic vesicles closely apposed to a postsynaptic density, begin to appear in the rodent visual cortex at P5–7 [84–86] (Fig. 3). Axonal and dendritic processes can be visualized at an ultrastructural level at earlier time points, but they do not show synaptic specializations until P5 [84, 87]. At this time cortical astrocytes are still dividing [63], have an immature morphology [16], and are expressing the synapse promoting factors, thrombospondins (Thbs) and glypicans (Gpc) [17, 18, 88] (Figs. 2 and 4). Some synapses can be visualized in the prenatal period, but these are mainly localized in the cortical preplate and will not be discussed here further [84, 85, 89–91].

Coincident with synapse formation, the majority of neuronal and astrocytic synapse associated proteins are beginning to be expressed during the first postnatal week in the cortex, peaking towards the end of the second postnatal week and then either subsiding or remaining stable to adulthood. These include proteins associated with presynaptic vesicle transport and release, postsynaptic density related proteins and neurotransmitter receptors of both excitatory and inhibitory synapses, as well as astrocyte-secreted synapse regulating proteins (Fig. 4). Interestingly, different family members for some synaptic proteins show divergent temporal expression patterns in the cortex, suggesting roles for particular family members in specific stages or types of synapse formation. This is also true for some of the astrocyte synapse-related proteins. Some examples from both cell types are outlined below:

1. Immunoreactivity for the vesicular glutamate transporter VGlut1, which marks presynaptic terminals of excitatory cortical neurons, is low at birth, but steadily increases with maturation. In contrast VGlut2 immunoreactivity is already high at P0 and peaks at P7, and then remains constant into adulthood [92–94] (Fig. 4). Contrary to VGlut1, cortical neurons do not express VGlut2 mRNA, and the protein immunoreactivity detected in the cortex is from presynaptic terminals of thalamic neurons, making their connections with cortical layers IV and I. Thus, VGluts mark pre-synaptic terminals from different sources, providing a way to distinguish these two types of circuits [94].

2. The postsynaptic glutamate receptors, N-methyl-D-aspartate and α-amino-3-hydroxy-5-methyl-4-isoxazolepropionic acid receptors (NMDARs; AMPARs), are crucial mediators of synaptic activity at excitatory glutamatergic synapses. Upon release of glutamate from presynaptic terminals it binds and activates AMPARs on the postsynaptic membrane, which will cause membrane depolarization and subsequent activation of NMDARs, leading to stabilization and potentiation of the synapse. Synapses that lack AMPAR mediated transmission (but contain NMDARs) are termed silent synapses [95], since at resting membrane potential NMDARs are blocked by Mg^2+^, which can be removed to activate the receptor upon membrane depolarization. It has been shown that silent synapses are more common during early postnatal development, and can be converted to active synapses with time [95]. AMPARs and NMDARs are composed of different subunits at different stages of postnatal development [96]. The GluA1 subunit of AMPARs peaks during the first postnatal week and then remains constant to adulthood [97, 98], while GluA2 subunit expression significantly increases later in development, around P14, coincident with synapse maturation [98, 99] (Figs. 2 and 4). NMDAR subunits are also developmentally regulated. GluN1 and GluN2B subunits are present at high levels at all ages examined, while the GluN2A subunit is low at birth and peaks during the second postnatal week [100–102]. At this time a developmental switch occurs for both types of receptors, where AMPAR subunit composition changes to include GluA2 [99], and NMDAR subunit composition changes from GluN2B containing to GluN2A containing receptors [101]. The different subunits vary in their functional properties: GluA2 subunit containing AMPARs are impermeable to Ca^2+^ [103], while GluN2 subunits differ in current decay time and sensitivity to Mg^2+^ block [100, 104]. Thus, the divergent subunit composition alters the functional output of receptor activation and the subsequent signaling pathways activated in the postsynaptic cell, influencing neuronal activity.

3. Astrocyte expression of Thbs1, Gpc4 and Gpc6 peaks in cortical astrocytes during the first postnatal week and is downregulated in the adult [88]. On the other hand expression of Hevin, another astrocyte-secreted synapse-promoting factor, is low at P1 in the cortex and superior colliculus (SC), peaks at P10–15 and stays high in adulthood [20, 22] (Fig. 4). This suggests that different astrocyte-secreted proteins may regulate the different stages of synaptogenesis i.e., initiation (first postnatal week) vs. maturation (second-third postnatal week; Fig. 2). Conversely, the astrocyte secreted specific inhibitor of Hevin, secreted protein acidic and rich in cysteine (SPARC), shows low expression in the SC at P10, peaks at P15, and is downregulated in the adult [20, 22].

The time course for GABAergic synapse development and synapse associated protein expression corresponds to that of excitatory synaptogenesis [85, 98, 105], with proteins associated with GABAergic presynaptic terminals such as vesicular GABA transporters (VGat) and the postsynaptic scaffolding protein Gephyrin following an overall similar developmental expression pattern as their excitatory synapse associated counterparts [98, 106] (Fig. 4). However, inhibitory circuit maturation occurs later in development, as at early stages GABA release leads to excitation of neurons due to a shift in the chloride ion equilibrium potential, and during the second postnatal week a shift from excitation to inhibition by GABA happens. At this time the excitatory/inhibitory balance, an important feature of normal brain developmental activity, is established [107]. The role of astrocytes in formation and function of inhibitory synapses has not been as extensively studied as excitatory synapse formation. Astrocytes express GABA receptors and transporters and respond to extracellular GABA [108–112]. In hippocampal and cortical neuron cultures astrocytes and astrocyte-secreted factors induce formation of inhibitory synapses [25, 27, 28]. Currently a role for astrocytes in inhibitory synapse formation in vivo, and the specific mechanisms by which astrocytes regulate inhibitory synapses, are largely unknown.

## Astrocyte secreted factors increase synaptic diversity

There are hundreds of different proteins that can be expressed at the neuronal synapse, which make up its molecular identity and are important for its formation and proper function. There are numerous cell adhesion molecules, components of transmitter release machinery, postsynaptic receptors and regulatory proteins such as neurexins, neuroligins, neural cell adhesion molecule (NCAM), protocadherins, receptor protein tyrosine phosphatases (RPTPs), leucine rich repeat transmembrane proteins (LRRTMs), tyrosine kinases (TrKs), ephrins and many more (reviewed in [113]). While some features are common to all synapses, such as the presence of neurotransmitter vesicles, many proteins are unique to a specific type of synapse or circuit. For example, an interaction between the postsynaptic neuroligin and presynaptic neurexin is present in both excitatory and inhibitory synapses, but the specific family member that is present varies [114]. Similarly, the different type IIa RPTPs (RPTPσ or RPTPδ) interact with several different targets to induce formation of either excitatory or inhibitory synapses [115]. The immense diversity of neuronal synaptic contacts stems from the specific interactions between distinct types of pre and postsynaptic proteins. But is that all? Or do astrocytes also contribute to synapse diversity? In this section we overview the molecular mechanisms of several astrocyte secreted proteins in the cortex, and how they may promote synaptic diversity.

As the number of known astrocyte secreted factors has grown, so has our knowledge of the diversity of their molecular mechanisms of action. However, whether a single astrocyte is expressing all synaptogenic factors, or if specialized astrocytes express a given synaptogenic factor or set of factors at the place and time a specific type of synapse or circuit is forming, is still unknown. Astrocyte secreted Hevin promotes formation of morphologically normal synapses that contain NMDARs but lack AMPARs (i.e. silent synapses), by binding and bridging a trans-synaptic connection between types of neurexin and neuroligin which otherwise don’t bind each other [20, 23]. Knock out of Hevin results in decreased expression of several critical postsynaptic proteins including postsynaptic density protein 95 (PSD95), Homer-1, the NMDAR subunits GluN1 and GluN2B, and the AMPAR GluA2 [23]. Furthermore, Hevin promotes formation of VGlut2 containing synapses in both the cortex and superior colliculus [20, 22], but has no effect on intracortical VGlut1 synapses [22]. Interestingly, astrocyte secreted Thbs also induces formation of silent VGlut2 containing synapses in the same brain regions [17, 19], but are expressed at earlier time points than Hevin (Fig. 4), suggesting Thbs may be involved in synapse initiation, and Hevin may be more important for maturation of these synapses. Thbs induces synapse formation via a different mechanism than Hevin, by signaling through the neuronal α2δ1 gabapentin receptor [19]. Overexpression of α2δ1 in the developing cortex promotes the formation of VGlut2 synapses, with no effect on VGlut1 [19], again demonstrating pathway-specific effects of astrocyte synaptogenic signals.

Astrocyte-secreted Gpc4 and 6 promote formation of active synapses by recruiting GluA1 AMPARs to nascent synaptic contact sites [18, 99] (Fig. 4). The early time point of Gpcs expression, together with their specific effect on recruiting GluA1 subunits of AMPARs which are found at immature synapses [116], points towards a role for Gpcs in synapse initiation. Interestingly, astrocytes or astrocyte conditioned media can recruit all subunits of AMPARs to the synapse [18], suggesting that astrocytes secrete additional factors that recruit GluA2 AMPARs and subsequent synapse maturation. These putative factors remain to be identified, and once known will provide yet another piece of information regarding the complex pattern of astrocyte-synapse regulation. The synaptogenic mechanism of Gpc4 involves the interaction of soluble Gpc4 with presynaptic RPTPδ and RPTPσ receptors, which induces the secretion of the AMPAR clustering factor Neuronal Pentraxin 1, promoting functional synapse formation. This demonstrates that the mechanism of action of Gpc4 is distinct from that of both Thbs and Hevin, which induce silent synapse formation [21]. Furthermore, RPTPσ is necessary to mediate the effects of both Thbs1 and Gpc4 in synapse formation (silent for Thbs1, active for Gpc4), while RPTPδ is specific to the Gpc4 pathway, further demonstrating the diversity of synaptogenic pathways that can be mediated through the same receptor by different astrocyte-derived proteins. Similar to Hevin and Thbs, Gpc4 is important for the formation of thalamocortical synapses, however its role at intracortical synapses is not known [21]. As Gpc4 and 6 show divergent expression patterns in the cortex during development [18], with Gpc6 being enriched in the upper cortical layers where VGlut1 synapses are present, it would be interesting to test whether Gpc4 and 6 differentially regulate VGlut2 vs VGlut1 synapses. In addition, SPARC specifically inhibits Hevin-mediated silent synapse formation [20] and also inhibits AMPAR recruitment to synapses [117], providing yet another layer of complexity to the distinct pathways by which astrocytes regulate synapse development. In future more research is needed to identify novel astrocyte-secreted factors that can influence other types of synapses such as GABAergic, cholinergic or dopaminergic.

## Development of astrocyte-synapse contact – What holds them together?

Studies focusing on astrocyte contact with excitatory synapses have shown that the amount of synapses contacted (or ensheathed) by astrocytes varies between brain regions from about 60–90% of the synapses in the cerebellum [118], 90% of synapses in the barrel cortex [119], 50%–90% of the synapses in the hippocampus [6, 120] and 80% of the synapses in the striatum [120](for review see also [121]). It was further shown that these contacts are dynamic and can be altered by neuronal activity, where an increase in neuronal activity leads to an increase in the extent of astrocyte coverage of dendritic spines, as well as an increase in the number of spines contacted by astrocyte processes [119, 122], while knocking out VGlut1 in the cortex results in fewer contacts between astrocytes and synapses, suggesting an overall role for glutamate signaling in this process [65].

Despite strong evidence from EM studies that astrocytes closely enwrap synapses, what holds the astrocyte process together with the synapse at the molecular level is not fully understood. Evidently, contact between astrocytes and neurons is important for synapse formation, as neurons cultured from E17 rat embryos, a time before astrocytes are generated, do not form synapses in response to astrocyte-secreted signals, while neurons cultured from later time points (E19), which have had previous contact with astrocytes, do [15]. Astrocytes express several known cell adhesion molecules including neuroligins, ephrins, and protocadherins [88], however unlike their well-established roles in providing the structural scaffold that holds together the pre- and postsynaptic sites, their role in anchoring the astrocyte process to the synapse during development are just beginning to be unraveled. Disruption of the eph-ephrin pathway in astrocytes during synapse development in the hippocampus led to a decrease in the lifetime of newly formed dendritic protrusions, suggesting that contact between developing dendrites and astrocyte processes are important for further dendritic stabilization [13]. Astrocytes in the spinal cord interact with neurons via γ-protocadherins, and knocking out their expression in astrocytes results in delayed synaptogenesis [123]. Finally, a recent study found that astrocytes in the visual cortex express the cell adhesion molecule neuroligin and contact neurons by binding to its well characterized partner, neurexin. Knocking out neuroligins in astrocytes caused a decrease in astrocyte morphological complexity and synaptic contact, and altered synaptic activity [16]. Therefore it seems astrocytes and neurons use similar scaffolding proteins to contact each other. Future studies are needed to reveal new astrocyte-synapse scaffolding proteins and further elucidate the mechanisms by which astrocyte-synapse contacts develop, for example by looking at different types of synapses and brain regions.

## Astrocyte signaling at the developing synapse – More ways than one

Astrocytes are non-electrically excitable cells, and they use several different signaling pathways to influence synapse formation and function, both during development and in adults. In addition to secreting synaptogenic proteins as discussed above, astrocytes express a variety of neurotransmitter receptors [124] which are activated by neurotransmitters released from adjacent neurons. One prominent form of astrocyte response to neurotransmitters is through elevation in intracellular calcium levels [122, 125, 126]. Increases in astrocyte calcium lead in some cases to the release of transmitter molecules such as glutamate, ATP or GABA, termed gliotransmitters that in turn, modulate astrocyte and neuronal activity [30, 80, 127]. Astrocyte calcium responses differ between their fine processes and the soma, suggesting that astrocytes can differentially react to the activity of individual synapses which contact their fine processes, as well as more globally to populations of cells [34, 128–130]. While it was shown that glutamate release by neurons can promote contact between astrocyte process and the synapse [65, 119], the effects of neuronal activity and transmitter release on astrocyte secretion of synaptogenic factors and whether this involves changes in intracellular calcium is unknown. To fully understand the complex role of astrocytes in synapse development, it is important in the future to investigate the mechanisms of astrocyte signaling pathways in the context of development and how it relates to synapse formation and function.

## Conclusions

Much progress has been made in understanding the role of astrocytes in the development of neuronal synapse structure and function, yet many open questions remain. Why are some synapses contacted by astrocytes and others are not? Does astrocyte-synapse contact change with development? Are astrocytes specialized to regulate specific synaptic connections? Future studies looking at more developmental time points, brain regions, synapse types as well as astrocyte heterogeneity are needed to provide a better understanding of synaptic development as a multicellular process.

## Abbreviations

AMPAR: α-amino-3-hydroxy-5-methyl-4-isoxazolepropionic receptor
CGE: Caudal ganglionic eminence
dLGN: Dorsal lateral geniculate nucleus
E: Embryonic day
EM: Electron microscopy
GABA: γ-aminobutyric acid
GFAP: Glial fibrillary acidic protein
Gpc: Glypican
LRRTM: Leucine rich repeat transmembrane proteins
MGE: Medial ganglionic eminence
NCAM: Neural Cell Adhesion Molecule
NF1A: Nuclear factor 1A
NMDAR: N-methyl-D-aspartate receptor
P: Postnatal day
PSD95: Postsynaptic density protein 95
RG: Radial glia
RPTP: Receptor protein tyrosine phosphatases
SC: Superior colliculus
SPARC: Secreted protein acidic and rich in cysteine
SVZ: Subventricular zone
Thbs: Thrombospondin
TrK: Tyrosine kinase
VGat: Vesicular GABA transporter
VGlut1, 2: Vesicular glutamate transporter 1, 2
VZ: Ventricular zone

## References

[1] Rakic P, Lombroso PJ (1998). Development of the cerebral cortex: I. Forming the cortical structure. J Am Acad Child Adolesc Psychiatry.

[2] Harris KD, Mrsic-Flogel TD (2013). Cortical connectivity and sensory coding. Nature.

[3] Douglas RJ, Martin KAC (2004). Neuronal circuits of the neocortex. Annu Rev Neurosci.

[4] Colón-Ramos DA (2009). Chapter 2 synapse formation in developing neural circuits. Curr Top Dev Biol.

[5] Gray EG (1959). Axo-somatic and axo-dendritic synapses of the cerebral cortex: an electron microscope study. J Anat.

[6] Ventura R, Harris KM (1999). Three-dimensional relationships between hippocampal synapses and astrocytes. J Neurosci.

[7] Araque A, Parpura V, Sanzgiri RP, Haydon PG (1999). Tripartite synapses: glia, the unacknowledged partner. Trends Neurosci.

[8] Barres BA (2008). The mystery and magic of glia: a perspective on their roles in health and disease. Neuron.

[9] Allen NJ (2014). Astrocyte regulation of synaptic behavior. Annu Rev Cell Dev Biol.

[10] Jäkel S, Dimou L (2017). Glial cells and their function in the adult brain: a journey through the history of their ablation. Front Cell Neurosci.

[11] Meyer-Franke A, Kaplan MR, Pfieger FW, Barres BA (1995). Characterization of the signaling interactions that promote the survival and growth of developing retinal ganglion cells in culture. Neuron.

[12] Ullian EM, Sapperstein SK, Christopherson KS, Barres BA (2001). Control of synapse number by glia. Science.

[13] Nishida H, Okabe S (2007). Direct astrocytic contacts regulate local maturation of dendritic spines. J Neurosci.

[14] Hama H, Hara C, Yamaguchi K, Miyawaki A (2004). PKC signaling mediates global enhancement of excitatory synaptogenesis in neurons triggered by local contact with astrocytes. Neuron.

[15] Barker AJ, Koch SM, Reed J, Barres BA, Ullian EM (2008). Developmental control of synaptic receptivity. J Neurosci.

[16] Stogsdill JA, Ramirez J, Liu D, Kim YH, Baldwin KT, Enustun E, Ejikeme T, Ji R-R, Eroglu C (2017). Astrocytic neuroligins control astrocyte morphogenesis and synaptogenesis. Nature.

[17] Christopherson KS, Ullian EM, Stokes CCA, Mullowney CE, Hell JW, Agah A, Lawler J, Mosher DF, Bornstein P, Barres BA (2005). Thrombospondins are astrocyte-secreted proteins that promote CNS synaptogenesis. Cell.

[18] Allen NJ, Bennett ML, Foo LC, Wang GX, Chakraborty C, Smith SJ, Barres BA (2012). Astrocyte glypicans 4 and 6 promote formation of excitatory synapses via GluA1 AMPA receptors. Nature.

[19] Eroglu Ç, Allen NJ, Susman MW, O'Rourke NA, Park CY, Özkan E, Chakraborty C, Mulinyawe SB, Annis DS, Huberman AD (2009). Gabapentin receptor α2δ-1 is a neuronal thrombospondin receptor responsible for excitatory CNS synaptogenesis. Cell.

[20] Kucukdereli H, Allen NJ, Lee AT, Feng A, Ozlu MI, Conatser LM, Chakraborty C, Workman G, Weaver M, Sage EH (2011). Control of excitatory CNS synaptogenesis by astrocyte-secreted proteins Hevin and SPARC. Proc Natl Acad Sci.

[21] Farhy-Tselnicker I, van Casteren ACM, Lee A, Chang VT, Aricescu AR, Allen NJ (2017). Astrocyte-secreted Glypican 4 regulates release of neuronal Pentraxin 1 from axons to induce functional synapse formation. Neuron.

[22] Risher WC, Patel S, Kim IH, Uezu A, Bhagat S, Wilton DK, Pilaz LJ, Singh Alvarado J, Calhan OY, Silver DL, et al. Astrocytes refine cortical connectivity at dendritic spines. elife. 2014;3 10.7554/eLife.04047 PMC4286724 25517933

[23] Singh SK, Stogsdill JA, Pulimood NS, Dingsdale H, Kim YH, Pilaz LJ, Kim IH, Manhaes AC, Rodrigues WS, Pamukcu A (2016). Astrocytes assemble Thalamocortical synapses by bridging NRX1alpha and NL1 via Hevin. Cell.

[24] Diniz LP, Almeida JC, Tortelli V, Vargas Lopes C, Setti-Perdigão P, Stipursky J, Kahn SA, Romão LF, de Miranda J, Alves-Leon SV (2012). Astrocyte-induced synaptogenesis is mediated by transforming growth factor β signaling through modulation of d-serine levels in cerebral cortex neurons. J Biol Chem.

[25] Diniz LP, Tortelli V, Garcia MN, Araújo APB, Melo HM, Seixas da Silva GS, De Felice FG, Alves-Leon SV, de Souza JM, Romão LF (2014). Astrocyte transforming growth factor beta 1 promotes inhibitory synapse formation via CaM kinase II signaling. Glia.

[26] Fuentes-Medel Y, Ashley J, Barria R, Maloney R, Freeman M, Budnik V (2012). Integration of a retrograde signal during synapse formation by glia-secreted TGF-β ligand. Curr Biol.

[27] Hughes EG, Elmariah SB, Balice-Gordon RJ (2010). Astrocyte secreted proteins selectively increase hippocampal GABAergic axon length, branching, and synaptogenesis. Mol Cell Neurosci.

[28] Elmariah SB, Oh EJ, Hughes EG, Balice-Gordon RJ (2005). Astrocytes regulate inhibitory synapse formation via Trk-mediated modulation of postsynaptic GABA<sub>a</sub> receptors. J Neurosci.

[29] Mauch DH, Nägler K, Schumacher S, Göritz C, Müller EC, Otto A, Pfrieger FW (2001). CNS synaptogenesis promoted by glia-derived cholesterol. Science.

[30] Papouin T, Dunphy J, Tolman M, Foley JC, Haydon PG. Astrocytic control of synaptic function. Philos Trans R Soc Lond Ser B Biol Sci. 2017;372(1715). 10.1098/rstb.2016.0154 PMC5247586 28093548

[31] Pannasch U, Rouach N. Emerging role for astroglial networks in information processing: from synapse to behavior. Trends Neurosci. 2013;36(7):405–17. 10.1016/j.tins.2013.04.004 23659852

[32] Bosworth AP, Allen NJ (2017). The diverse actions of astrocytes during synaptic development. Curr Opin Neurobiol.

[33] Baldwin KT, Eroglu C (2017). Molecular mechanisms of astrocyte-induced synaptogenesis. Curr Opin Neurobiol.

[34] Khakh BS, Sofroniew MV (2015). Diversity of astrocyte functions and phenotypes in neural circuits. Nat Neurosci.

[35] Clancy B, Darlington RB, Finlay BL (2001). Translating developmental time across mammalian species. Neuroscience.

[36] Gilmore EC, Herrup K (1997). Cortical development: layers of complexity. Curr Biol.

[37] Molyneaux BJ, Arlotta P, Menezes JRL, Macklis JD. Neuronal subtype specification in the cerebral cortex. Nat Rev Neurosci. 2007;8:427. 10.1038/nrn2151 17514196

[38] Rash BG, Grove EA (2006). Area and layer patterning in the developing cerebral cortex. Curr Opin Neurobiol.

[39] Sur M, Rubenstein JL (2005). Patterning and plasticity of the cerebral cortex. Science.

[40] Chaboub LS, Deneen B (2012). Developmental origins of astrocyte heterogeneity: the final frontier of CNS development. Dev Neurosci.

[41] Freeman MR. Specification and morphogenesis of astrocytes. Science. 2010;330(6005):774–8. 10.1126/science.1190928 PMC5201129 21051628

[42] Miller FD, Gauthier AS (2007). Timing is everything: making neurons versus glia in the developing cortex. Neuron.

[43] Kanski R, van Strien ME, van Tijn P, Hol EM (2014). A star is born: new insights into the mechanism of astrogenesis. Cell Mol Life Sci.

[44] Sloan SA, Barres BA (2014). Mechanisms of astrocyte development and their contributions to neurodevelopmental disorders. Curr Opin Neurobiol.

[45] Rowitch DH, Kriegstein AR (2010). Developmental genetics of vertebrate glial–cell specification. Nature.

[46] Molofsky AV, Deneen B (2015). Astrocyte development: a guide for the perplexed. Glia.

[47] Kriegstein A, Alvarez-Buylla A (2009). The glial nature of embryonic and adult neural stem cells. Annu Rev Neurosci.

[48] Greene NDE, Copp AJ (2009). Development of the vertebrate central nervous system: formation of the neural tube. Prenat Diagn.

[49] Noctor SC, Martínez-Cerdeño V, Ivic L, Kriegstein AR (2004). Cortical neurons arise in symmetric and asymmetric division zones and migrate through specific phases. Nat Neurosci.

[50] Gao P, Postiglione Maria P, Krieger Teresa G, Hernandez L, Wang C, Han Z, Streicher C, Papusheva E, Insolera R, Chugh K (2014). Deterministic progenitor behavior and unitary production of neurons in the neocortex. Cell.

[51] Beattie R, Hippenmeyer S (2017). Mechanisms of radial glia progenitor cell lineage progression. FEBS Lett.

[52] Götz M, Hartfuss E, Malatesta P (2002). Radial glial cells as neuronal precursors: a new perspective on the correlation of morphology and lineage restriction in the developing cerebral cortex of mice1 1Please note the highly relevant work by Miyata et al., 2001, that appeared after submission of this article. The work by Miyata and colleagues demonstrates beautifully the asymmetric division of radial glial cells with one daughter transforming into a neuron as suggested in our article. Brain Res Bull.

[53] Rakic P (1972). Mode of cell migration to the superficial layers of fetal monkey neocortex. J Comp Neurol.

[54] Lopez-Bendito G, Molnar Z (2003). Thalamocortical development: how are we going to get there?. Nat Rev Neurosci.

[55] Sahara S, Yanagawa Y, O’Leary DDM, Stevens CF (2012). The fraction of cortical GABAergic neurons is constant from near the start of cortical neurogenesis to adulthood. J Neurosci.

[56] Markram H, Toledo-Rodriguez M, Wang Y, Gupta A, Silberberg G, Wu C (2004). Interneurons of the neocortical inhibitory system. Nat Rev Neurosci.

[57] Kepecs A, Fishell G (2014). Interneuron cell types are fit to function. Nature.

[58] Huang ZJ (2014). Toward a genetic dissection of cortical circuits in the mouse. Neuron.

[59] Namihira M, Kohyama J, Semi K, Sanosaka T, Deneen B, Taga T, Nakashima K. Committed neuronal precursors confer astrocytic potential on residual neural precursor cells. Dev Cell. 2009;16(2):245–55. 10.1016/j.devcel.2008.12.014 19217426

[60] Bonni A, Sun Y, Nadal-Vicens M, Bhatt A, Frank DA, Rozovsky I, Stahl N, Yancopoulos GD, Greenberg ME (1997). Regulation of Gliogenesis in the central nervous system by the JAK-STAT signaling pathway. Science.

[61] He F, Ge W, Martinowich K, Becker-Catania S, Coskun V, Zhu W, Wu H, Castro D, Guillemot F, Fan G (2005). A positive autoregulatory loop of Jak-STAT signaling controls the onset of astrogliogenesis. Nat Neurosci.

[62] Kang P, Lee HK, Glasgow SM, Finley M, Donti T, Gaber ZB, Graham BH, Foster AE, Novitch BG, Gronostajski RM (2012). Sox9 and NFIA coordinate a transcriptional regulatory Cascade during the initiation of Gliogenesis. Neuron.

[63] Ge W-P, Miyawaki A, Gage FH, Jan YN, Jan LY (2012). Local generation of glia is a major astrocyte source in postnatal cortex. Nature.

[64] Bandeira F, Lent R, Herculano-Houzel S (2009). Changing numbers of neuronal and non-neuronal cells underlie postnatal brain growth in the rat. Proc Natl Acad Sci U S A.

[65] Morel L, Higashimori H, Tolman M, Yang Y (2014). VGluT1(+) neuronal glutamatergic signaling regulates postnatal developmental maturation of cortical protoplasmic Astroglia. J Neurosci.

[66] Bushong EA, Martone ME, Jones YZ, Ellisman MH (2002). Protoplasmic astrocytes in CA1 stratum radiatum occupy separate anatomical domains. J Neurosci.

[67] Bushong EA, Martone ME, Ellisman MH (2004). Maturation of astrocyte morphology and the establishment of astrocyte domains during postnatal hippocampal development. Int J Dev Neurosci.

[68] Halassa MM, Fellin T, Takano H, Dong J-H, Haydon PG (2007). Synaptic Islands defined by the territory of a single astrocyte. J Neurosci.

[69] Buosi AS, Matias I, Araujo APB, Batista C, Gomes FCA. Heterogeneity in Synaptogenic Profile of Astrocytes from Different Brain Regions. Mol Neurobiol. 2017;55(1):751–62. 10.1007/s12035-016-0343-z 28050794

[70] Emsley JG, Macklis JD (2006). Astroglial heterogeneity closely reflects the neuronal-defined anatomy of the adult murine CNS. Neuron Glia Biol.

[71] John Lin C-C, Yu K, Hatcher A, Huang T-W, Lee HK, Carlson J, Weston MC, Chen F, Zhang Y, Zhu W (2017). Identification of diverse astrocyte populations and their malignant analogs. Nat Neurosci.

[72] Morel L, Chiang MSR, Higashimori H, Shoneye T, Iyer LK, Yelick J, Tai A, Yang Y (2017). Molecular and functional properties of regional astrocytes in the adult brain. J Neurosci.

[73] Eilam R, Aharoni R, Arnon R, Malach R (2016). Astrocyte morphology is confined by cortical functional boundaries in mammals ranging from mice to human. elife.

[74] Houades V, Koulakoff A, Ezan P, Seif I, Giaume C (2008). Gap junction-mediated astrocytic networks in the mouse barrel cortex. J Neurosci.

[75] Schipke CG, Haas B, Kettenmann H (2008). Astrocytes discriminate and selectively respond to the activity of a subpopulation of neurons within the barrel cortex. Cereb Cortex.

[76] Harris KM, Weinberg RJ (2012). Ultrastructure of synapses in the mammalian brain. Cold Spring Harb Perspect Biol.

[77] Yook C, Druckmann S, Kim J (2013). Mapping mammalian synaptic connectivity. Cell Mol Life Sci.

[78] Ippolito DM, Eroglu C (2010). Quantifying synapses: an immunocytochemistry-based assay to quantify synapse number. J Vis Exp.

[79] Wouterlood FG, Böckers T, Witter MP (2003). Synaptic contacts between identified neurons visualized in the confocal laserscanning microscope. Neuroanatomical tracing combined with immunofluorescence detection of post-synaptic density proteins and target neuron-markers. J Neurosci Methods.

[80] Martín R, Bajo-Grañeras R, Moratalla R, Perea G, Araque A (2015). Circuit-specific signaling in astrocyte-neuron networks in basal ganglia pathways. Science.

[81] Perea G, Yang A, Boyden ES, Sur M. Optogenetic astrocyte activation modulates response selectivity of visual cortex neurons in vivo. Nat Commun. 2014;5:3262. 10.1038/ncomms4262 PMC4075037 24500276

[82] Chaudhry FA, Lehre KP, Lookeren Campagne M, Ottersen OP, Danbolt NC, Storm-Mathisen J. Glutamate transporters in glial plasma membranes: highly differentiated localizations revealed by quantitative ultrastructural immunocytochemistry. Neuron. 1995;15(3):711–20. 10.1016/0896-6273(95)90158-2 7546749

[83] Higashi K, Fujita A, Inanobe A, Tanemoto M, Doi K, Kubo T, Kurachi Y (2001). An inwardly rectifying K+ channel, Kir4.1, expressed in astrocytes surrounds synapses and blood vessels in brain. Am J Phys Cell Phys.

[84] Li M, Cui Z, Niu Y, Liu B, Fan W, Yu D, Deng J (2010). Synaptogenesis in the developing mouse visual cortex. Brain Res Bull.

[85] Blue ME, Parnavelas JG (1983). The formation and maturation of synapses in the visual cortex of the rat. I. Qualitative analysis. J Neurocytol.

[86] Blue ME, Parnavelas JG (1983). The formation and maturation of synapses in the visual cortex of the rat. II. Quantitative analysis. J Neurocytol.

[87] Miller M, Peters A (1981). Maturation of rat visual cortex. II. A combined Golgi-electron microscope study of pyramidal neurons. J Comp Neurol.

[88] Cahoy JD, Emery B, Kaushal A, Foo LC, Zamanian JL, Christopherson KS, Xing Y, Lubischer JL, Krieg PA, Krupenko SA (2008). A transcriptome database for astrocytes, neurons, and oligodendrocytes: a new resource for understanding brain development and function. J Neurosci.

[89] König N, Roch G, Marty R (1975). The onset of synaptogenesis in rat temporal cortex. Anat Embryol.

[90] Li SP, Lee HY, Park MS, Bahk JY, Chung BC, Kim MO (2006). Prenatal GABAB1 and GABAB2 receptors: cellular and subcellular organelle localization in early fetal rat cortical neurons. Synapse.

[91] Verhage M, Maia AS, Plomp JJ, Brussaard AB, Heeroma JH, Vermeer H, Toonen RF, Hammer RE, van den Berg TK (2000). Synaptic assembly of the brain in the absence of neurotransmitter secretion. Science.

[92] Boulland J-L, Qureshi T, Seal RP, Rafiki A, Gundersen V, Bergersen LH, Fremeau RT, Edwards RH, Storm-Mathisen J, Chaudhry FA (2004). Expression of the vesicular glutamate transporters during development indicates the widespread corelease of multiple neurotransmitters. J Comp Neurol.

[93] Minelli A, Edwards RH, Manzoni T, Conti F (2003). Postnatal development of the glutamate vesicular transporter VGLUT1 in rat cerebral cortex. Brain Res Dev Brain Res.

[94] Nakamura K, Hioki H, Fujiyama F, Kaneko T (2005). Postnatal changes of vesicular glutamate transporter (VGluT)1 and VGluT2 immunoreactivities and their colocalization in the mouse forebrain. J Comp Neurol.

[95] Hanse E, Seth H, Riebe I (2013). AMPA-silent synapses in brain development and pathology. Nat Rev Neurosci.

[96] Traynelis SF, Wollmuth LP, McBain CJ, Menniti FS, Vance KM, Ogden KK, Hansen KB, Yuan H, Myers SJ, Dingledine R (2010). Glutamate receptor ion channels: structure, regulation, and function. Pharmacol Rev.

[97] Martin LJ, Furuta A, Blackstone CD (1998). AMPA receptor protein in developing rat brain: glutamate receptor-1 expression and localization change at regional, cellular, and subcellular levels with maturation. Neuroscience.

[98] Gonzalez-Lozano MA, Klemmer P, Gebuis T, Hassan C, van Nierop P, van Kesteren RE, Smit AB, Li KW (2016). Dynamics of the mouse brain cortical synaptic proteome during postnatal brain development. Sci Rep.

[99] Brill J, Huguenard JR (2008). Sequential changes in AMPA receptor targeting in the developing neocortical excitatory circuit. J Neurosci.

[100] Monyer H, Burnashev N, Laurie DJ, Sakmann B, Seeburg PH (1994). Developmental and regional expression in the rat brain and functional properties of four NMDA receptors. Neuron.

[101] Sanz-Clemente A, Matta JA, Isaac JTR, Roche KW (2010). Casein kinase 2 regulates the NR2 subunit composition of synaptic NMDA receptors. Neuron.

[102] Sheng M, Cummings J, Roldan LA, Jan YN, Jan LY (1994). Changing subunit composition of heteromeric NMDA receptors during development of rat cortex. Nature.

[103] Kumar SS, Bacci A, Kharazia V, Huguenard JR (2002). A developmental switch of AMPA receptor subunits in neocortical pyramidal neurons. J Neurosci.

[104] Kutsuwada T, Kashiwabuchi N, Mori H, Sakimura K, Kushiya E, Araki K, Meguro H, Masaki H, Kumanishi T, Arakawa M (1992). Molecular diversity of the NMDA receptor channel. Nature.

[105] De Felipe J, Marco P, Fairén A, Jones EG (1997). Inhibitory synaptogenesis in mouse somatosensory cortex. Cereb Cortex.

[106] Pinto JGA, Jones DG, Murphy KM (2013). Comparing development of synaptic proteins in rat visual, somatosensory, and frontal cortex. Front Neural Circuits.

[107] Zhang Z, Jiao Y-Y, Sun Q-Q (2011). Developmental maturation of excitation and inhibition balance in principal neurons across four layers of somatosensory cortex. Neuroscience.

[108] Kang J, Jiang L, Goldman SA, Nedergaard M (1998). Astrocyte-mediated potentiation of inhibitory synaptic transmission. Nat Neurosci.

[109] Fraser DD, Mudrick-Donnon LA, Macvicar BA (1994). Astrocytic GABA receptors. Glia.

[110] Gould T, Chen L, Emri Z, Pirttimaki T, Errington AC, Crunelli V, Parri HR. GABA<sub>B</sub> receptor-mediated activation of astrocytes by gamma-hydroxybutyric acid. Philos Trans R Soc B. 2014;369(1654) 10.1098/rstb.2013.0607 PMC4173292 25225100

[111] Meier SD, Kafitz KW, Rose CR (2008). Developmental profile and mechanisms of GABA-induced calcium signaling in hippocampal astrocytes. Glia.

[112] Perea G, Gómez R, Mederos S, Covelo A, Ballesteros JJ, Schlosser L, Hernández-Vivanco A, Martín-Fernández M, Quintana R, Rayan A (2016). Activity-dependent switch of GABAergic inhibition into glutamatergic excitation in astrocyte-neuron networks. elife.

[113] McAllister AK (2007). Dynamic aspects of CNS synapse formation. Annu Rev Neurosci.

[114] Graf ER, Zhang X, Jin S-X, Linhoff MW, Craig AM (2004). Neurexins induce differentiation of GABA and glutamate postsynaptic specializations via Neuroligins. Cell.

[115] Takahashi H, Craig AM (2013). Protein tyrosine phosphatases PTPδ, PTPσ, and LAR: presynaptic hubs for synapse organization. Trends Neurosci.

[116] Henley JM, Wilkinson KA (2016). Synaptic AMPA receptor composition in development, plasticity and disease. Nat Rev Neurosci.

[117] Jones EV, Bernardinelli Y, Tse YC, Chierzi S, Wong TP, Murai KK (2011). Astrocytes control glutamate receptor levels at developing synapses through SPARC–β-integrin interactions. J Neurosci.

[118] Xu-Friedman MA, Harris KM, Regehr WG (2001). Three-dimensional comparison of ultrastructural characteristics at depressing and facilitating synapses onto cerebellar Purkinje cells. J Neurosci.

[119] Genoud C, Quairiaux C, Steiner P, Hirling H, Welker E, Knott GW (2006). Plasticity of astrocytic coverage and glutamate transporter expression in adult mouse cortex. PLoS Biol.

[120] Chai H, Diaz-Castro B, Shigetomi E, Monte E, Octeau JC, Yu X, Cohn W, Rajendran PS, Vondriska TM, Whitelegge JP (2017). Neural circuit-specialized astrocytes: transcriptomic, proteomic, morphological, and functional evidence. Neuron.

[121] Bernardinelli Y, Muller D, Nikonenko I (2014). Astrocyte-synapse structural plasticity. Neural Plasticity.

[122] Bernardinelli Y, Randall J, Janett E, Nikonenko I, König S, Jones EV, Flores CE, Murai KK, Bochet CG, Holtmaat A (2014). Activity-dependent structural plasticity of perisynaptic astrocytic domains promotes excitatory synapse stability. Curr Biol.

[123] Garrett AM, Weiner JA (2009). Control of CNS synapse development by γ-Protocadherin-mediated astrocyte–neuron contact. J Neurosci.

[124] Porter JT, McCarthy KD (1997). Astrocytic neurotransmitter receptors in situ and in vivo. Prog Neurobiol.

[125] Sun W, McConnell E, Pare J-F, Xu Q, Chen M, Peng W, Lovatt D, Han X, Smith Y, Nedergaard M. Glutamate-dependent neuroglial calcium signaling differs between young and adult brain. Science. 2013;339(6116):197–200. 10.1126/science.1226740 PMC3569008 23307741

[126] López-Hidalgo M, Schummers J (2014). Cortical maps: a role for astrocytes?. Curr Opin Neurobiol.

[127] Martin-Fernandez M, Jamison S, Robin LM, Zhao Z, Martin ED, Aguilar J, Benneyworth MA, Marsicano G, Araque A. Synapse-specific astrocyte gating of amygdala-related behavior. Nat Neurosci. 2017;20:1540. 10.1038/nn.4649 PMC5903286 28945222

[128] Srinivasan R, Huang BS, Venugopal S, Johnston AD, Chai H, Zeng H, Golshani P, Khakh BS (2015). Ca2+ signaling in astrocytes from Ip3r2−/− mice in brain slices and during startle responses in vivo. Nat Neurosci.

[129] Di Castro MA, Chuquet J, Liaudet N, Bhaukaurally K, Santello M, Bouvier D, Tiret P, Volterra A (2011). Local Ca2+ detection and modulation of synaptic release by astrocytes. Nat Neurosci.

[130] Zheng K, Bard L, Reynolds JP, King C, Jensen Thomas P, Gourine Alexander V, Rusakov Dmitri A. Time-resolved imaging reveals heterogeneous landscapes of Nanomolar ca^2+^ in neurons and Astroglia. Neuron. 2015;88(2):277–88. 10.1016/j.neuron.2015.09.043 PMC4622934 26494277

[131] Romand S, Wang Y, Toledo-Rodriguez M, Markram H (2011). Morphological development of thick-tufted layer V pyramidal cells in the rat somatosensory cortex. Front Neuroanat.

[132] Berton F, Iborra C, Boudier J-A, Seagar MJ, Marquèze B (1997). Developmental regulation of Synaptotagmin I, II, III, and IV mRNAs in the rat CNS. J Neurosci.

[133] Brakeman PR, Lanahan AA, O'Brien R, Roche K, Barnes CA, Huganir RL, Worley PF (1997). Homer: a protein that selectively binds metabotropic glutamate receptors. Nature.

[134] Zurmöhle U-M, Herms J, Schlingensiepen R, Schlingensiepen K-H, Brysch W (1994). Changes of synapsin I messenger RNA expression during rat brain development. Exp Brain Res.

[135] Bogen IL, Jensen V, Hvalby O, Walaas SI (2009). Synapsin-dependent development of glutamatergic synaptic vesicles and presynaptic plasticity in postnatal mouse brain. Neuroscience.

[136] Minelli A, Alonso-Nanclares L, Edwards RH, DeFelipe J, Conti F (2003). Postnatal development of the vesicular gaba transporter in rat cerebral cortex. Neuroscience.

